# Substance dependence: Decades apart in a teaching hospital

**DOI:** 10.4103/0019-5545.42396

**Published:** 2008

**Authors:** J. Venkatesan, Stelina S. D. Suresh

**Affiliations:** Department of Psychiatry, Thanjavur Medical College, Thanjavur, Tamilnadu, India

**Keywords:** Alcohol dependence, comorbidity, polysubstance dependence

## Abstract

**Aim:**

The present study was done to understand the changing trends in substance dependence across decades.

**Settings and Design:**

It is a retrospective study done in Department of Psychiatry in a Teaching Hospital setting. The data of patients who attended the OPD for substance dependence during the months January to December in the years 1985 & 1986, 1995 & 1996 and 2005 & 2006 were collected and analysed.

**Materials and Methods:**

A total of 839 new patients with substance dependence identified according to International Classification of Diseases (ICD) (*n* = 839) was analysed in the present report. Study variables taken into account are alcohol dependence, polysubstance dependence which also includes alcohol, age, sex, age of initiation of substance use, duration of use, and comorbidity.

**Statistical Analysis:**

*Z*-test, Chi-square test, mean, percentages, standard deviation.

**Results:**

Substance dependence constituted 5.32% in 1985 and 1986, 5.02% in 1995 and 1996, and 4.05% in 2005 and 2006 of the newly registered total psychiatric patients. The variation in incidence figures across the years is statistically not significant (*P* > 0.05). Among the substance dependents 2% in 1985 & 1986, 1% in 1995 & 1996 and 1% in 2005 & 2006 were females. Majority of the patients were alcohol dependent (87.2% in 1985 and 1986, 89.4% in 1995 and 1996, and 79.6% in 2005 and 2006). Polysubstance dependence showed an increasing trend and it was statistically significant. Comparison of the years 1985 and 1986 with 2005 and 2006 gives *Z* = 2.4, *P* < 0.05 (statistically significant). Comparison of the years 1995 and 1996 with 2005 and 2006 gives *Z* = 3, *P* < 0.01 (significant statistically). Number of people getting initiated to substance use in early age (*viz.* 10-19 years) showed an increasing trend. People with positive family history of substance dependence started using substances early in life. (Chi-square value: 164.7, *P* < 0.0001, significant statistically). In polysubstance dependence comorbidity was more (*Z* = 4.1, *P* < 0.001, significant statistically).

**Conclusions:**

Incidence of substance dependence remained the same across the two decades. But incidence of polysubstance dependence is increasing over the years. People start using substances earlier and are becoming dependent earlier in their lives in the present decade. Polysubstance dependence is correlated with greater comorbidity. Early recognition of comorbidity and its management is essential for better prognosis. Substance dependence is exclusively a male diagnosis in our population.

## INTRODUCTION

Substance dependence is a psychiatric disorder, characterized by compulsive substance use and appearance of withdrawal symptoms when the substance is no longer used.[1] Alcohol-related disorders affect 5-10% of world population each year. Incidence and prevalence of alcohol-related problems are believed to be increasing[2,3] as the society becomes westernized and we get globalized. Newer substances of abuse are encountered[2] in our day-to-day clinical practice. Hence, the present study was done with an aim to understand the changing trends in substance dependence across decades.

## SUBJECTS AND METHODS

This is a retrospective study conducted in Psychiatry Department of a teaching Hospital. The data of the patients who attended psychiatry OPD for substance dependence during the months January to December in the years 1985 and 1986, 1995 and 1996, and 2005 and 2006 was collected and analysed. A total of 839 cases of substance dependence was identified (*n* = 839) according to International Classification of Diseases (ICD)[4,5] and studied. Statistical analysis was done using *Z* test, chi-square test, mean, percentages, and standard deviation. Study variables taken into account are the alcohol use, polysubstance use, age, sex, age of initiation of substance use, duration of use, and comorbidity.

## RESULTS

### Incidence

A total of 344 new patients with substance dependence were registered in 1985 and 1986, 245 in 1995 and 1996, and 250 in 2005 and 2006 (total - 839 patients). Substance dependence constitutes 5.3% in 1985 and 1986, 5% in 1995 and 1996, and 4.5% in 2005 and 2006 of the total psychiatric population attending OPD during these years. Incidence across decades does not show much variation and is not significant statistically.

### Sex

Females constitute 2% in 1985 and 1986 and 1% in 1995 and 1996 and 1% in 2005 and 2006 of substance dependence. Out of the total 344 substance dependent cases, seven females were encountered in 1985 and 1986, and two out of 245 and 250 cases in 1995 and 1996 and 2005 and 2006. No significant variation is noted across years. The male:female ratio is 120:1. All the substance dependent females had co morbid illness and the co morbidity among female substance dependents is 100%.The commonest co morbidity noted was mood disorder. A family member with substance abuse had great impact on substance dependence in females. Of the eleven female substance dependents ten had their significant family members also dependent on substances.

### Age

Mean age of patients with drug dependence is 38.2 years in 1985 and 1986, 39.1 years in 1995 and 1996, and 38.4 years in 2005 and 2006. No significant variation in age is noted across decades [Table 1].

**Table 1:** Age and year-wise classification

	1985	1986	1995	1996	2005	2006
10-19		3	1		4	
20-29	41	28	22	22	23	24
30-39	62	74	52	37	47	43
40-49	58	38	49	31	38	44
50-59	22	11	9	17	7	16
60-69	3	2	-	5	1	3
70-79	-	2	-	-		
Total	186	158	133	112	120	130
Mean	38.8	36.8	38.2	38.7	37.0	39.6
SD	10.1	9.2	8.6	9.5	9.6	10.1
*P*	*P* > 0.05		*P* > 0.05		*P* > 0.05	
Combined mean	38.2		39.1		38.4	
SD	10.1		9.8		9.9	
*P*	*P* > 0.05		*P* > 0.05		*P* > 0.05	

Mean age of patients coming to treatment - 38 years. *P* value not significant.

### Comparison of alcohol and polysubstance dependence

**Figure 1:**
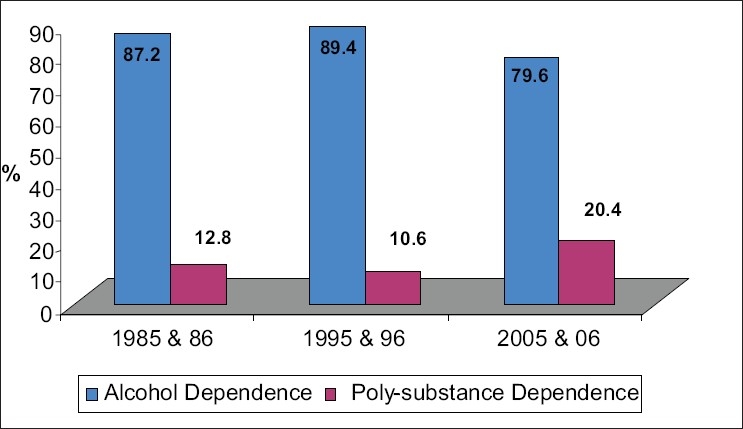
Comparison of alcohol and polysubstance dependence. Comparison of polysubstance dependence in 85 & 86 with 2005 & 2006 *Z* = 2.4, *P*<0.05, significant statistically. Comparison of polysubstance dependence in 95 & 96 with 2005 & 2006 *Z* = 3, *P* < 0.01 significant statistically. Polysubstance dependence shows increasing trend over recent years (05 & 06).

Majority are alcohol dependent. 87.2% in 1985 and 1986, 89.4% in 1995 and 1996, and 79.6% in 2005 and 2006 .Of the total 344 substance dependent cases in 1985 and 1986, 300 were alcohol dependent and 44 polysubstance dependent. Of the 245 cases encountered in 1995 and 1996, 219 were alcohol dependent and 26 polysubstance dependent and in 2005 and 2006 out of total 250 cases, 199 were alcohol dependent and 51 were polysubstance dependent. The alarming point to note is that the number of patients with polysubstance dependence is increasing in the present decade (05 & 06) and this is statistically significant. Polysubstance dependence is 12.8% in 1985 and 1986, 10.6% in 1995 and 1996, and 20.4% in 2005 and 2006. Comparing the years 1985 and 1986 with 2005 and 2006 gives the value of *P* < 0.05 and this is significant statistically [Figure 1]. Comparison of years 1995 and 1996 with 2005 and 2006 shows *P* < 0.01 and again this is significant statistically.

### Polysubstance dependence

**Figure 2:**
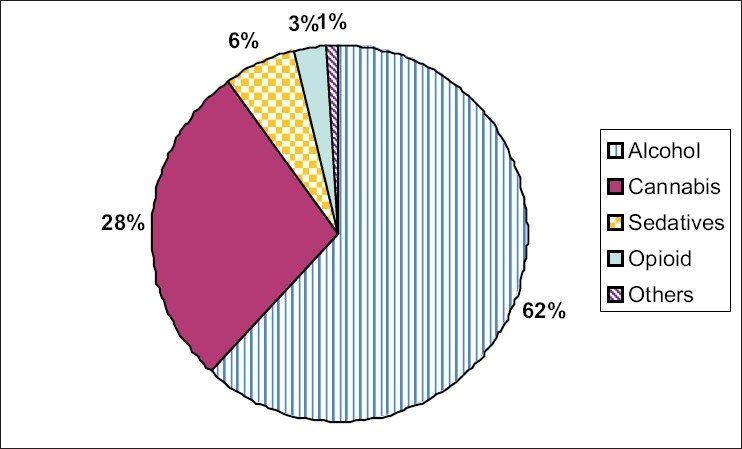
Pie chart showing polysubstance dependence

Among polysubstance dependence alcohol forms the major substance of dependence in 62% followed by cannabis in 28%, sedatives 6%, and opioids in 3%. The most frequent substance combination encountered was that of alcohol and cannabis. The combination was seen in 61 out of 121 polysubstance dependents (50%). Opioid dependence was not seen in the present years (2005 and 2006). Among the four opioid dependent patients encountered in 85 & 86, two were using raw opium and two were using heroin. Heroin and newer psychotropic substances were not found in the recent years. Amphetamine dependence was not encountered in the study [Figure 2].

### Age of initiation of substance use

**Figure 3:**
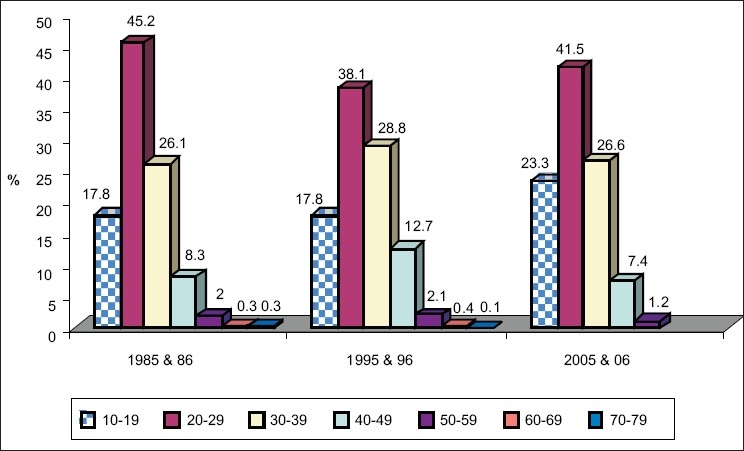
Age of initiation and year-wise classification. Shows increase in age group (10-19 years) across the years Z = 1.54 *P* <0.05 not significant

There is a rising trend in number of people who get initiated to substance use in early age (10-19 years) In the recent years (05 & 06). In the age group 10-19 years 17.8% is seen in 1985 and 1986 and 1995 and 1996, and 23.3% in 2005 and 2006 [Figure 3].

### Age of initiation and positive family history of substance dependence

Substance usage occurs early in those who have positive family history of substance dependence. Of the total 188 people with positive family history of substance dependence, 91 took to substances in 10-19 years (*P* < 0.0001, significant statistically) [Figure 4].

**Figure 4(a):**
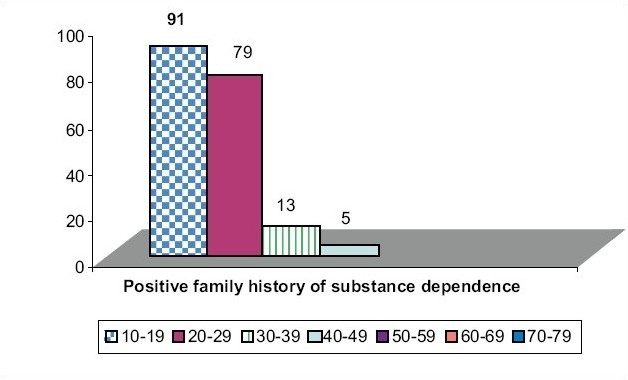
Age of initiation and positive family history of substance dependence. Initiation of substance abuse in 10-19 years with positive family history chi-square value = 164.7, *P*<0.0001, Significant statistically. People with positive family history of substance dependence take substances earlier

**Figure 4(b):**
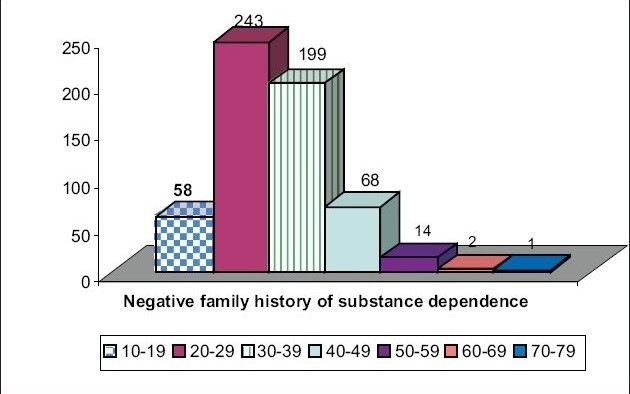
Age of initiation and negative family history of substance dependence

### Substance dependence and comorbidity

Accurate diagnosis and differentiation between substance induced states and primary illness is one of the most difficult tasks. In the study to differentiate this three points were taken into consideration-onset of psychiatric symptoms before onset of substance abuse, family history of particular psychiatric disorder and sustained psychiatric symptoms during lengthy periods of abstinence. These details collected from the data were used in the study. The findings showed a similar trend across the decades; hence significant findings in all the three periods together are discussed.

Comorbid psychiatric illness is noted in 33.3% of alcohol dependents and 54.5% of polysubstance dependent patients. Physical comorbidity is noted in 4.9% of alcohol dependents and 2.5% of polysubstance dependents. Mood disorder is the commonest comorbid condition noted in 21.5% of polysubstance dependents and 21.6% of alcohol dependents. Similarly schizophrenia is noted in 24.8% of polysubstance dependents and 4.3% of alcohol dependents (*P* < 0.001, significant statistically). Personality disorder is noted in 7.4% of polysubstance dependent patients and 2.6% in alcohol dependent patients (*P* < 0.05, significant statistically). 3.8% of alcohol dependent patients had comorbid anxiety disorders while 0.8% polysubstance dependent patients had the same (*P* < 0.01, significant statistically). Results confirm the fact that in polysubstance dependence comorbidity is more and this is significant statistically (*P* < 0.001). Mood disorder is the commonest comorbid illness. Polysubstance dependence is more in schizophrenia and personality disorder [Table 2].

**Table 2:** Substance dependence and co morbidity

Comorbidity	1985 and 1986		1995 and 1996		2005 and 2006		Total			
				
	Total	A	Ps.D	A	Ps.D	A	A Total	A %	Ps.D Total	Ps.D %
Personality disorder	13	2	3	2	3	5	19	2.6	9	7.4
Mood disorder	61	10	39	2	55	14	155	21.6	26	21.5
Schizophrenia	12	8	11	7	8	15	**31**	**4.3**	**30**	**24.8**
Anxiety disorders	10	1	10	-	7	-	27	3.8	1	0.8
Medical disorder	16	1	9	2	10	-	35	4.9	3	2.5
No comorbidity	188	22	147	13	116	17	**451**	**62.8**	**52**	**42.9**
Total	300	44	219	26	199	51	**718**	**-**	**121**	**-**

^*^Alcohol dependence with comorbidity 42.8; Polysubstance dependence with comorbidity 57.1. ^*^In polysubstance dependence comorbidity is more. *Z* = 4.1 *P* < 0.001, significant statistically. ^*^Schizophrenics tend to use polysubstance rather than alcohol alone. *Z* = 5.12, *P* < 0.001, significant statistically.

## DISCUSSION

Our study gives the pattern of substance dependence over a period of two decades. In our study 98% of substance dependents were males. Majority of the patients were alcohol dependent (87.2% in 85 & 86, 79.6% in 05 & 06). We did not come across amphetamine dependence in our study. A retrospective study by Adamson *et al.*[6] compares the various variables of a substance dependent population from a geographical area in New Zealand for the years 1998 and 2004 (6 years interval). They noted 65% substance dependents were males. Alcohol-related problems were seen in 47% and cannabis in 24%, and opioid in 15% of patients. Amphetamine dependence was found to increase from 0% in 1998 to 10% in 2004. Sachdev *et al.*[7] compared the changing pattern of substance dependence between the years 1994 and1998 (four years interval) among patients attending Deaddiction centre at Faridkat. They found people are moving towards more novel substances. But we could not observe this in the population around our area. Heroin and newer psychotropic substances dependence could not be found in our local population.

Substances dependence is rare in females. Male: female ratio is 120:1 in our study. All our substance dependent females had co morbid illness, co morbidity being 100% in female substance dependents. Of the total eleven female substance dependents ten had either their husband or family members also dependent on substances. In a retrospective study by Adamson *et al.*[6] in New Zealand, 35% substance dependents were females. The comparative figures are significantly lower in India. Willsnack *et al.*[8] report from United States of America shows 60% of all women and 70% of men in the general population take alcohol. This reflects the fact that substance dependence is still mainly a problem of males in our cultural setup and there may be reluctance in the part of females to come for treatment.[9,10] Alcoholic women are also more stigmatized.[11] Women are more likely to face multiple barriers to accessing substance abuse treatment and less likely to seek treatment.[12] They tend to seek treatment in primary care setting rather than speciality treatment.[12,13] Women are found to progress more from substance use to dependence (telescoping effect)[14] and they develop more physical and psychological complications.[15] This is confirmed by our study findings. A study by Brady *et al.*[16] shows women are mostly influenced by boyfriends or parents, or partners who are already dependent on substances. Findings of our study are similar.

Polysubstance dependence shows increasing trend in recent years. In our study there is rise in number of persons taking to substances earlier in their life in the present decade and this correlates with the worldwide trend.[2,17–22] Recent trends in substance abuse in China shows a re-emergence of substance abuse (increasing trend in substances abuse) and also abuse of new substances.[17] Fournier *et al.*[22] gives an overall slight decline in tobacco, methamphetamine, heroin, and club drinks. Though America is congratulating itself in curbing the alcohol and illicit substance use and the decline in teen smoking, abuse and addiction of controlled prescription substances, i.e., opioid, CNS depressants, and stimulants have been stealthily but sharply rising[2] in the United States. The teenage abuse of prescription substances has increased from 5.4% in 2002 to 6.3% in 2005.[2] Wilson *et al.*[23] states that clinical impression of adolescent substance abuse underestimates the problem and calls for structured screening devices. Early age of use of alcohol and early regular drinking is a marker of risk for later dependence.[20,21]

An abuser of psychotropic substance in a house has tremendous impact on others in the family. In our study people with positive family history of substance dependence start using substances earlier in their lives and it is significant statistically (*P* < 0.0001). This correlates with the study done by Kadri *et al.*[24] in a Deaddiction Center in Ahmedabad. People with positive family history of alcohol dependence have higher prevalence of lifetime alcohol dependence.[25] Risk of initiation of alcohol use prior to 15 years of age is greater in those with family history of alcohol dependence.[26] Genetic and environmental factors play a part in this association.[26,27] Paternal maximum alcohol consumption predicts substance abuse or dependence in male and female offsprings.[28]

In our study alcohol dependence with comorbidity is 37.2% and polysubstance dependents have comorbidity of 57%. It correlates well with Epidemiological Catchment Area (ECA) study[29] in which alcohol dependents with comorbid mental disorder is 37% and polysubstance dependents have comorbid mental disorder of 53%. In National Comorbidity Survey (NCS) study,[30] 78% men and 86% women with alcohol use had comorbid mental disorders. In our study, mood disorder is the commonest comorbid condition associated with substance dependence (21.6%). The ECA study[29] and National Epidemiologic Survey on Alcohol and Related Conditions (NESARC)[31] reports odds ratio between substance use disorder and any mood disorder greater than odds ratio for other conditions. Recent findings from NESARC[31] show greater association for mood disorder across eight specific substance use disorders.[32] In contrast NCS[30] reported associations between substance dependence and any anxiety disorder exceed that for any mood disorder. In ECA study, bipolar disorder was the most common axis I condition likely to occur in substance use disorder.[29,33] Schizophrenics tend to be dependent on polysubstance.[34,35] High prevalence of substance abuse is present in schizophrenics.[36] In our study, comorbid schizophrenia was diagnosed in 4.3% of alcohol dependent cases and 24.8% in polysubstances dependent cases. This is significant statistically. Of the total 61 cases diagnosed as schizophrenia 30 patients (50%) were polysubstance dependent cases. Schizophrenics tend to be polysubstance dependent rather than alcohol alone *P*< 0.001. This correlates with the study by Verdoux *et al.*[37] in which nearly half, i.e., 47.8% of substance dependents with comorbid schizophrenia, schizo affective, bipolar disorder were dependent on at least two different substances. Study by Green *et al.*[38] and Winklbaur *et al.*[36] shows that schizophrenics are dependent on polysubstance mostly alcohol, cannabis, nicotine, and cocaine. Second to alcohol, cannabis is most frequently misused substance in a patient with schizophrenia.[39] Rate of comorbid substance dependence in schizophrenia is three times higher than in the general population.[40]

The clinical impact of comorbidity is substantial. Comorbid disorders worsen the prognosis of treatment and increase use of services and health care costs.[32,33] The treatment of comorbid substance use and mental disorders can be challenging for the clinician and the health care system.[31,33] Careful evaluation of the symptoms of each disorder is required followed by formulation of an integrated treatment plan.[32]

## CONCLUSION

Alcoholic beverages have been used in human societies since time immemorial. Cultural differences apparently influence pattern of alcohol consumption.[41] Substance dependence is a multidimensional problem.[42] It involves not only the person, but also influences the society on diverse ways. Contrary to our expectation we did not see a rising trend in substance dependence among patients attending psychiatry department in our study across decades. But polysubstance usage is increasing. The other alarming fact is more number of people are getting initiated to substance use earlier in their life.[2,17–21] Awareness for early treatment and early diagnosis of comorbid diseases is necessary and this has major implications for management.[31–33,43] Family members should be role model for youngsters. We have to adapt methods to prevent polysubstance use.
